# Proliferation of latently infected CD4^+^ T cells carrying replication-competent HIV-1: Potential role in latent reservoir dynamics

**DOI:** 10.1084/jem.20170193

**Published:** 2017-04-03

**Authors:** Nina N. Hosmane, Kyungyoon J. Kwon, Katherine M. Bruner, Adam A. Capoferri, Subul Beg, Daniel I.S. Rosenbloom, Brandon F. Keele, Ya-Chi Ho, Janet D. Siliciano, Robert F. Siliciano

**Affiliations:** 1 Department of Medicine, Johns Hopkins University School of Medicine, Baltimore, MD 21205; 2 Department of Biomedical Informatics, Columbia University Medical Center, New York, NY 10032; 3 AIDS and Cancer Virus Program, Leidos Biomedical Research Inc., Frederick National Laboratory for Cancer Research, Frederick, MD 21702; 4 Howard Hughes Medical Institute, Baltimore, MD 21205

## Abstract

A latent reservoir for HIV-1 in resting CD4^+^ T lymphocytes precludes cure. Mechanisms underlying reservoir stability are unclear. Recent studies suggest an unexpected degree of infected cell proliferation in vivo. T cell activation drives proliferation but also reverses latency, resulting in productive infection that generally leads to cell death. In this study, we show that latently infected cells can proliferate in response to mitogens without producing virus, generating progeny cells that can release infectious virus. Thus, assays relying on one round of activation underestimate reservoir size. Sequencing of independent clonal isolates of replication-competent virus revealed that 57% had *env* sequences identical to other isolates from the same patient. Identity was confirmed by full-genome sequencing and was not attributable to limited viral diversity. Phylogenetic and statistical analysis suggested that identical sequences arose from in vivo proliferation of infected cells, rather than infection of multiple cells by a dominant viral species. The possibility that much of the reservoir arises by cell proliferation presents challenges to cure.

## Abstract (precis)

The latent reservoir for HIV-1 in resting CD4^+^ T cells prevents cure with antiretroviral therapy. Hosmane et al. provide evidence supporting the hypothesis that a larger fraction of cells in the reservoir is generated by cell proliferation than by direct infection.

## Introduction

A stable latent reservoir for HIV-1 in resting memory CD4^+^ T cells persists, despite antiretroviral therapy (ART; Chun et al., 1995, 1997a,b; Finzi et al., 1997, 1999; Wong et al., 1997; Siliciano et al., 2003; Strain et al., 2003; Crooks et al., 2015). The extremely long half-life of this reservoir is a major barrier to cure (Finzi et al., 1999; Siliciano et al., 2003; Strain et al., 2003; Crooks et al., 2015). This reservoir of latent but replication-competent HIV-1 was originally identified in resting CD4^+^ T cells in the blood and lymph node (Chun et al., 1995, 1997a), but known patterns of circulation, activation, and differentiation of memory T cells predict that persistent HIV-1 will reside in multiple memory cell subsets in multiple tissues (Chomont et al., 2009; Buzon et al., 2014; Soriano-Sarabia et al., 2014; Banga et al., 2016; Boritz et al., 2016). The latent reservoir is a major target of cure efforts, some of which focus on reversing latency so that infected cells can be eliminated by immune mechanisms (Richman et al., 2009; Archin et al., 2012; Halper-Stromberg et al., 2014; Deeks et al., 2016).

One potential explanation for the remarkable stability of the latent reservoir involves the proliferation of infected cells (Tobin et al., 2005; Bailey et al., 2006; Chomont et al., 2009; Bosque et al., 2011; Maldarelli et al., 2014; Wagner et al., 2014; Lorenzi et al., 2016; Simonetti et al., 2016). Proliferation of infected cells is to some extent unexpected. Some stimuli that drive T cell proliferation also drive latently infected cells into a productively infected state, and productively infected cells have a very short half-life (1 d; Ho et al., 1995; Wei et al., 1995). In addition, the HIV-1 Vpr protein causes cell cycle arrest (Jowett et al., 1995; Stewart et al., 1997, 2000; Sakai et al., 2006; DeHart et al., 2007; Hrecka et al., 2007; Schröfelbauer et al., 2007; Romani and Cohen, 2012). In some model systems, cytokines including IL-7 and IL-15 can drive homeostatic proliferation of CD4^+^ T cells without inducing virus gene expression (Bosque et al., 2011; Vandergeeten et al., 2013). However, IL-7 can also reverse latency in some systems (Scripture-Adams et al., 2002; Wang et al., 2005).

Despite these issues, there is considerable evidence that infected cells can proliferate in vivo. The evidence comes in two forms. In patients who start ART during chronic infection, the extensive viral sequence diversification that takes place before treatment (Shankarappa et al., 1999; Brodin et al., 2016) makes it unlikely that multiple independently sampled viral sequences from a single patient will be identical. Therefore, repeated isolation of identical viral sequences from individual patients can be most readily explained by assuming that an initially infected cell carrying the sequence subsequently proliferated, copying the integrated viral genome without error into progeny cells. Sequencing of trace levels of plasma virus present in treated patients initially provided the surprising result that this residual viremia was often dominated by a single frequently isolated sequence (Tobin et al., 2005; Bailey et al., 2006). Subsequent studies of proviral DNA also revealed independent identical sequences (Bailey et al., 2006; von Stockenstrom et al., 2015; Bruner et al., 2016; Lorenzi et al., 2016). Although these studies strongly suggest in vivo proliferation of infected cells, there are caveats. Isolates that are identical in the sequenced part of the genome may differ elsewhere and may not be clonal (Laskey et al., 2016) or may represent separate infection events with an identical virus. Furthermore, the vast majority of proviruses are defective (Ho et al., 2013; Bruner et al., 2016; Imamichi et al., 2016), and without full-genome sequencing (Ho et al., 2013) or viral outgrowth assays (VOAs; Lorenzi et al., 2016), it remains unclear whether the identical sequences represent replication-competent virus. An important recent study by Lorenzi et al. has examined a large number of independent isolates of replication-competent virus from treated patients and found that >50% share sequence identity in the *env* gene with other isolates from the same patients (Lorenzi et al., 2016).

Definitive evidence for the proliferation of infected cells has come from another experimental approach, the analysis of HIV-1 integration sites. Recent studies have demonstrated that, within a given patient, a surprisingly large fraction of infected CD4^+^ T cells shows proviral integration into precisely the same position in the human genome (Maldarelli et al., 2014; Wagner et al., 2014; Cohn et al., 2015). Given the relatively nonspecific integration of HIV-1 into expressed genes throughout the genome (Schröder et al., 2002; Han et al., 2004), this result can only be explained by the proliferation of infected cells after integration. Interestingly, some of the expanded clones showed integration into cellular genes associated with cell survival and/or proliferation, raising the possibility that altered host gene expression could drive proliferation (Maldarelli et al., 2014; Wagner et al., 2014). However, integration site analysis captures only the ends of the viral genome, and it is likely that most of the expanded cellular clones detected by integration site analysis are replication defective, as has been shown by Cohn et al. (2015). Unfortunately, with current methods, it is difficult to simultaneously and efficiently obtain both the integration site and the full proviral sequence. Interestingly, a recent study by Simonetti et al. (2016) has described a massively expanded CD4^+^ T cell clone carrying replication-competent virus in a patient with a complex disease course.

Collectively, these results raise the interesting possibility that the proliferation of infected cells may contribute to the stability of the latent reservoir. To explore this issue, we performed ex vivo stimulations and single-genome analysis to look for evidence of clonal expansion in infected individuals on ART.

## Results

### Analysis of infected-cell proliferation using a multiple stimulation VOA (MS-VOA)

**Figure 1.**
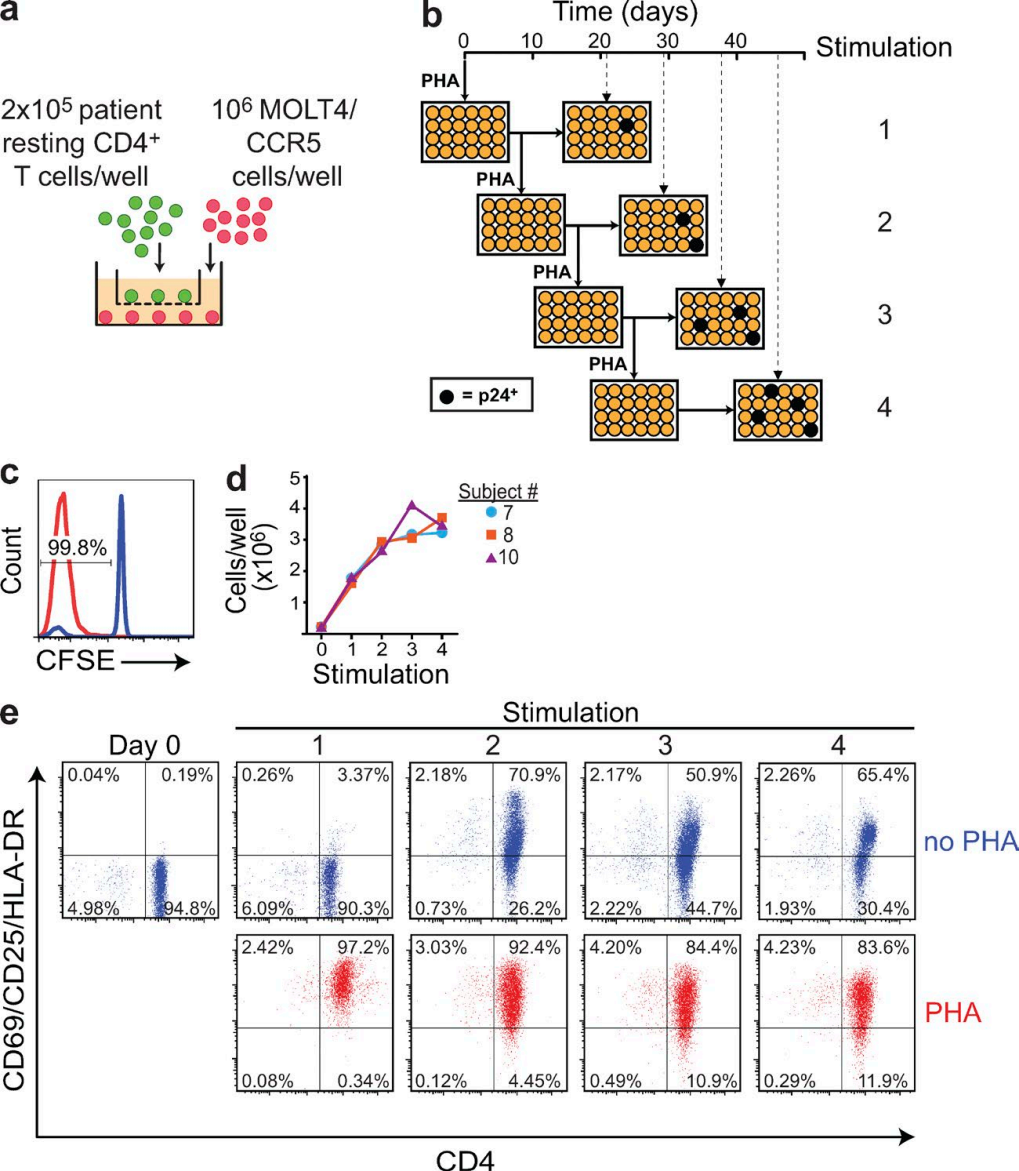
**MS-VOA.** (a) A transwell co-culture system (Ho et al., 2013) was used to allow separate manipulation of patient CD4^+^ T cells (green) and MOLT4/CCR5 cells (red). Purified resting CD4^+^ T cells from subjects on ART were plated at a limiting dilution for viral outgrowth and stimulated with PHA and irradiated allogeneic PBMCs (not depicted) as previously described (Finzi et al., 1997, 1999; Siliciano et al., 2003; Laird et al., 2016). After 24 h, PHA was removed, and 10^6^ MOTL4/CCR5 cells were added. Outgrowth in this system is equivalent to standard co-cultures (Ho et al., 2013). (b) Assay time course. At 8 d after the initial PHA stimulation, half the volume from the top and bottom chambers of each transwell was transferred to a new set of transwell plates for a second round of PHA stimulation. The initial plates were cultured without further stimulation for a total of 21 d. The restimulated plates were cultured for 8 d after the second round of PHA stimulation and then split as above to generate a third set of plates, which received a third round of stimulation. Similarly, these plates were split 8 d later to generate the fourth set of plates, which received a fourth round of stimulation. A p24 ELISA was performed 21 d after each respective round of PHA stimulation to quantify viral outgrowth (dashed lines). In this hypothetical example, each round of PHA stimulation induced outgrowth from an additional well (black circles). (c) PHA stimulation induces uniform proliferation of resting CD4^+^ T cells. Immediately before the first PHA stimulation, an aliquot of resting CD4^+^ T cells was stained with CFSE. CFSE dilution was quantitated by flow cytometry 7 d after stimulation to determine the fraction of cells that had proliferated (red histogram). Cells that did not receive PHA stimulation served as controls (blue histograms). The histogram is representative of CFSE dilution from three subjects. (d) Increase in cell number after each round of stimulation. Results for three representative subjects are shown. (e) Activation marker expression induced by each round of PHA stimulation. Cells were stained with antibodies to CD4 (x axis) and the activation markers CD25, CD69, and HLA-DR (y axis) 7 d after the indicated round of PHA stimulation (red dot plots). Cells that did not receive the most recent round of PHA stimulation were analyzed in parallel (blue dot plots). Results are shown for subject 10 and are representative of two other subjects analyzed.

Given that productively infected cells have a short in vivo half-life (1 d; Ho et al., 1995; Wei et al., 1995), we hypothesized that proliferation of latently infected cells carrying replication-competent HIV-1 could take place without release of infectious virus. To test this hypothesis, we subjected resting CD4^+^ T cells from patients on long-term ART to multiple rounds of mitogen stimulation to detect virus release from cells that had proliferated in response to a previous stimulation without producing infectious virus. This assay, modified from the standard quantitative VOA (QVOA; Chun et al., 1997a; Finzi et al., 1997, 1999; Siliciano et al., 2003; Siliciano and Siliciano, 2005; Laird et al., 2013, 2016) used to measure latently infected cells, is described in Fig. 1. Limiting dilutions of purified resting CD4^+^ T cells were maximally stimulated with the potent T cell mitogen PHA and irradiated allogeneic PBMCs from normal donors. CCR5-transfected MOLT4 cells (MOLT4/CCR5; Laird et al., 2013) were added to expand virus released from cells in which latency was reversed. Patient cells and MOLT4/CCR5 cells were plated in separate chambers of transwell plates. This allows separate manipulation of patient cells, also giving T cell activation and viral outgrowth equivalent to standard co-cultures (Ho et al., 2013). After 8 d, half of the contents of each well were transferred to the corresponding chambers of new plates, which received an additional stimulation. The original plates were cultured without the additional stimulation for a total of 21 d (Fig. 1 b). A total of four sequential stimulations were performed in this manner. Each well was cultured for 21 d after the most recent stimulation, sufficient time to allow virus released from a single cell to grow exponentially in MOLT4/CCR5 cells to levels readily detectable by p24 ELISA (Laird et al., 2013). Then, viral RNA in the supernatants of p24^+^ wells was subjected to sequence analysis. This protocol allows assessment of whether cells that have previously proliferated in response to T cell activation without producing infectious virus can release virus with additional stimulation (Fig. 1 b).

The initial stimulation caused >99% of patient resting CD4^+^ T cells to proliferate (Fig. 1 c). Cell number increased eightfold during the first week, with smaller increases after the second, third, and fourth stimulations (3.5-, 2.5-, and twofold, respectively; Fig. 1 d). The initial stimulation also caused >99% of the cells to express T cell activation markers (Fig. 1 e). Although cells did not return to a fully quiescent state before the second, third, and fourth stimulations, each stimulation increased activation marker expression relative to cells that did not receive the most recent stimulation (Fig. 1 e).

**Figure 2.**
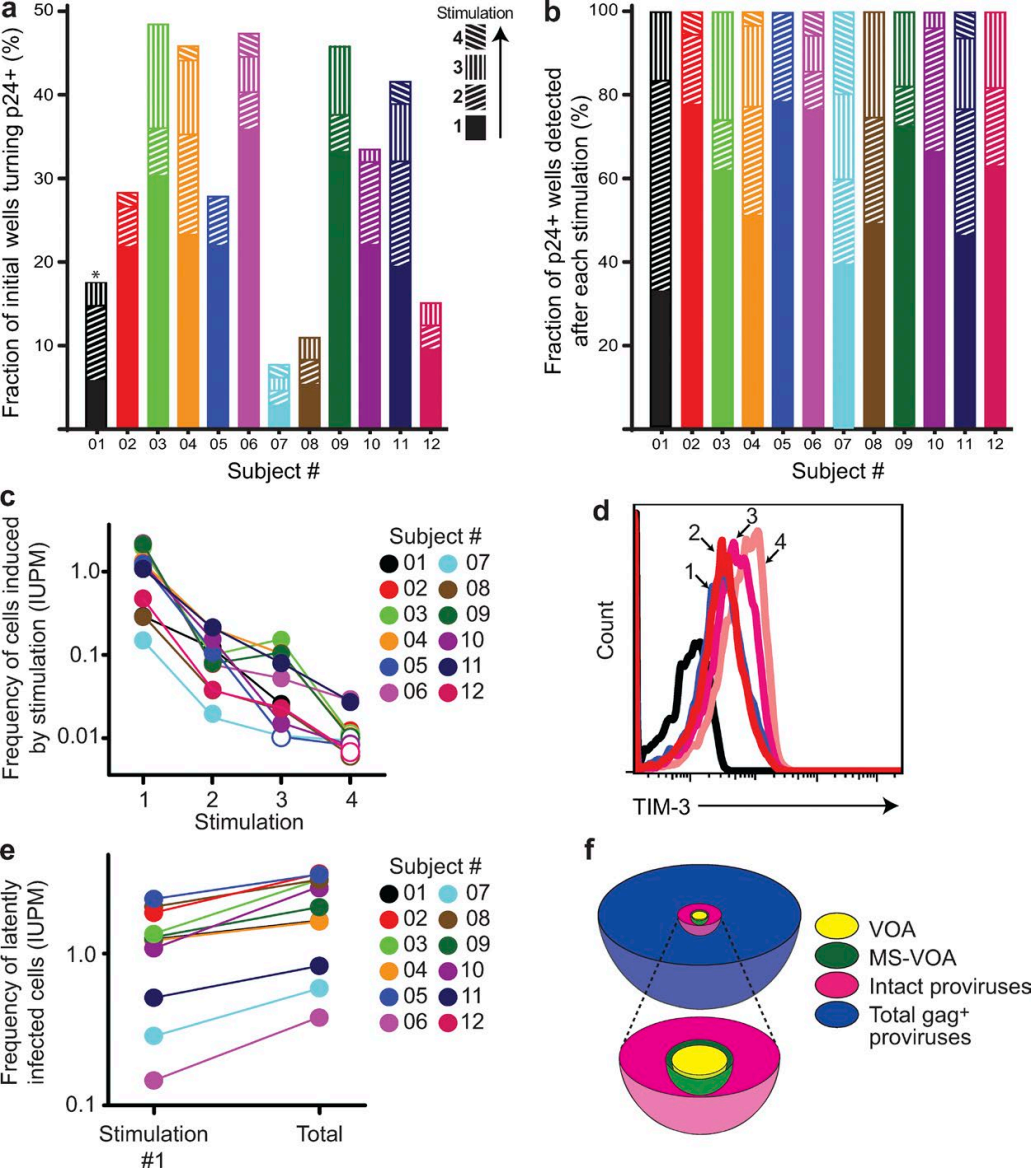
**Further rounds of T cell activation induce additional proviruses to produce replication-competent virus.** (a) Fraction of initially seeded wells becoming p24^+^ after each of four consecutive stimulations with PHA. For each subject (*n* = 12), a total of 64–72 wells were initially seeded. The asterisk indicates that cells from subject 1 only received three rounds of PHA stimulation. (b) Fraction of total p24^+^ wells from each subject (*n* = 12) that were detected for each round of PHA stimulation. The denominator is the total number of initially seeded wells. (c) Frequency of cells in the culture at the time of stimulation that are induced to produce replication-competent virus by the indicated round of stimulation. Frequency in infectious units per million (IUPM) cells was determined for each subject (*n* = 12) from mean cell counts before each stimulation and the number of wells turning p24^+^ after stimulation using a maximum likelihood estimation of infected cell frequency (Rosenbloom et al., 2015). (d) Expression of TIM-3 before stimulation (black line) and 7 d after the indicated round of stimulation. Results are presentative of exhaustion marker analysis in three subjects. (e) Frequency of latently infected cells among the initially plated cells from each subject (*n* = 12) as detected after a single round of PHA stimulation and after a total of four consecutive rounds of stimulation. (f) Schematic representation of the relative frequency of infected resting CD4^+^ T cells detected by different assays. The VOA result presents the mean frequency detected after a single round of PHA stimulation in cultures from 12 subjects studied here. The MS-VOA result represents the mean frequency among the initially plated cells of latently infected cells as detected by a total of four consecutive rounds of PHA stimulation. The frequencies of cells with intact (nondefective) proviruses and the total frequency of infected cells as measured by PCR with *gag* primers were estimated from the VOA results and the ratios described by Bruner et al. (2016). The true frequency of latently infected cells is between the frequency measured in the MS-VOA and the frequency of cells with intact proviruses.

This MS-VOA was performed on purified resting CD4^+^ T cells from 12 participants (01–12) on long-term suppressive ART (see Table S1 for patient characteristics). The mean frequency of latently infected cells detected after the first stimulation was 0.83 infectious units per million resting CD4^+^ T cells, not significantly different from that observed in studies using the standard QVOA (Finzi et al., 1999; Siliciano et al., 2003; Eriksson et al., 2013). However, for every subject, the additional rounds of stimulation caused viral outgrowth in cultures split from wells that remained negative for viral outgrowth without the additional stimulation (Fig. 2 a). Because all cells proliferate in response to PHA (Fig. 1 c), this result indicates that some latently infected cells proliferated without releasing infectious virus but retained the capacity to do so after subsequent stimulation. These results are consistent with previous work showing that a single round of stimulation in the standard QVOA does not detect all the latent virus present (Ho et al., 2013) and with a study in a primary cell model showing that cytokine-driven homeostatic proliferation of latently infected cells can occur without up-regulation of HIV-1 gene expression (Bosque et al., 2011).

These studies provided insight into the relationship between T cell activation, proliferation, and latency reversal. By splitting cultures of proliferating CD4^+^ T cells, it is possible to determine whether additional stimulation can induce virus production when other cells from the same clonal population fail to produce virus after the initial stimulation. Of all cultures that eventually become positive for outgrowth, only a mean of 60% were detected after the first stimulation (Fig. 2 b). In 11 of 12 patients, two rounds of stimulation were insufficient to induce all of the proviruses that were ultimately induced with the third or fourth round (Fig. 2, a and b). These results illustrate the difficulty in purging the latent reservoir even with maximum T cell activation. Multiple rounds of maximal stimulation are likely to be required. However, in 4 of 12 participants, 70–80% of isolates were obtained in the first round of stimulation. Interestingly, because cell number increased with each stimulation (Fig. 1 d), the per-cell probability of outgrowth fell after the first stimulation (Fig. 2 c). This may reflect differences in proviral inducibility, with a readily induced population and a generally smaller population that is more difficult to induce. Alternatively, changes in the transcriptional environment with repetitive stimulation during long-term in vitro culture might prevent induction of some intact proviruses that could be induced in vivo. To address this possibility, we examined CD4^+^ T cell expression of surface proteins associated with functional inhibition or immune exhaustion (Anderson et al., 2016) including PD-1, CTLA-4, and Tim-3. We observed increased Tim-3 expression after multiple stimulations (Fig. 2 d). Exhaustion of the cells in culture may contribute to the consecutive decreases in the probability of outgrowth. Thus, the MS-VOA provides only a minimal estimate of latent reservoir size because immune exhaustion developing with repetitive stimulation and/or long-term in vitro culture may not allow outgrowth from all potentially inducible replication-competent proviruses. The frequency of latently infected cells detected with the MS-VOA was about twofold greater than the standard QVOA value measured after one round of stimulation (Fig. 2 e). The actual frequency of latently infected cells is likely to lie between the MS-VOA measurement and the total number of intact (nondefective) proviruses (Fig. 2 f). Together, these results demonstrate that latently infected cells carrying replication-competent HIV-1 can proliferate in response to ex vivo stimulation without producing infectious virus, though retaining the ability to do so subsequently.

### Independent isolation of identical sequences of replication-competent virus from the latent reservoir

**Figure 3.**
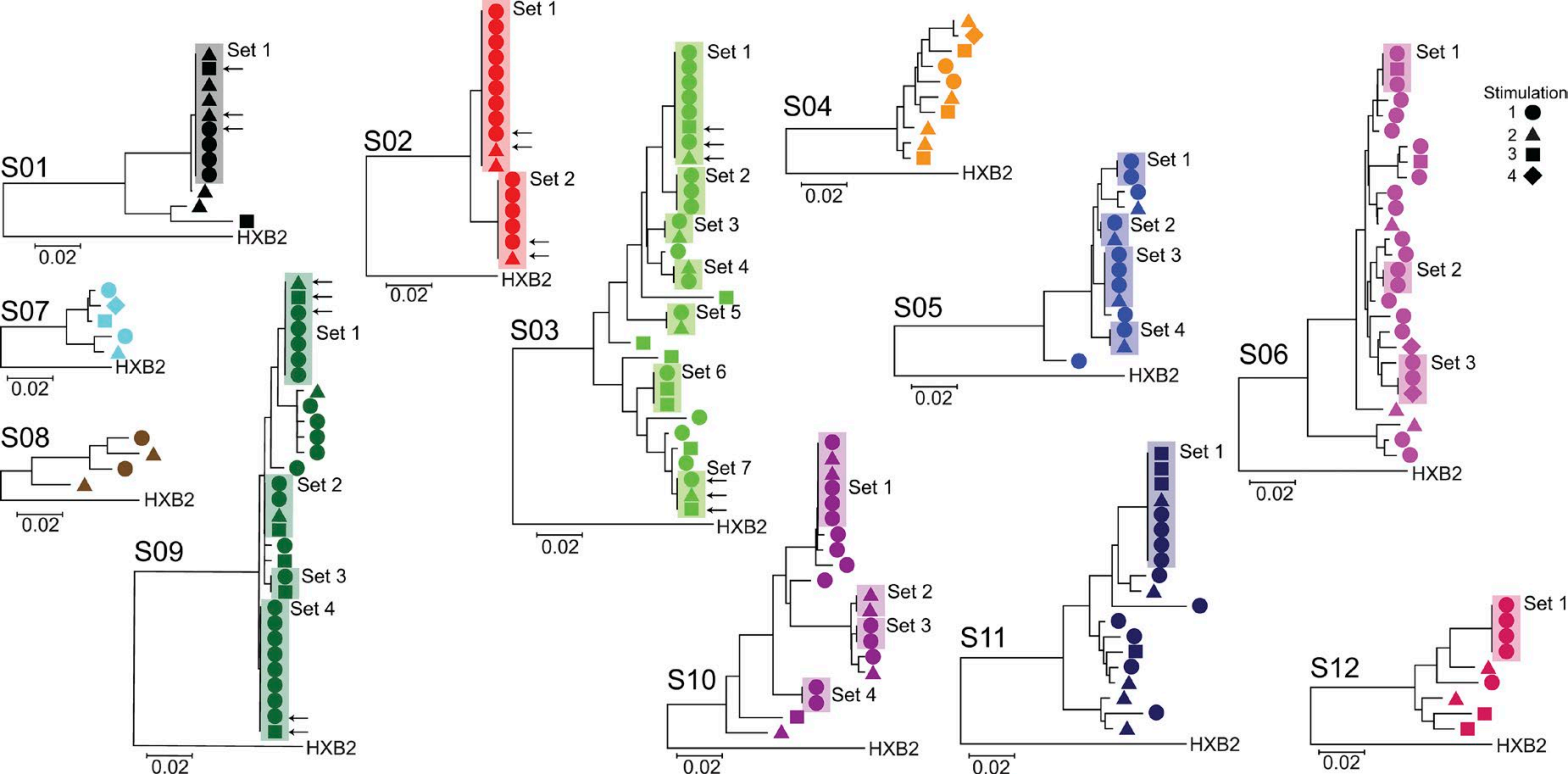
**Many independent isolates of replication-competent HIV-1 have identical sequences.** Phylogenetic trees of *env* sequences of independent isolates of replication-competent virus from each subject (*n* = 12). Sequencing was performed on genomic viral RNA in supernatants of p24^+^ wells. Cultures were established at limit dilution for viral outgrowth. Isolates containing more than a single sequence as indicated by double peaks at one or more positions in the chromatograms were not further analyzed. Symbols indicate the number of PHA stimulations after which the isolate was obtained. Sets of isolates with identical *env* sequences are boxed. Representative isolates obtained after different numbers of stimulations (arrows) from randomly selected sets were subjected to full-length sequencing at the single-genome level (see Fig. 4).

The ability of latently infected cells to proliferate ex vivo without releasing virus suggests that in vivo clonal expansion of infected cells could maintain the latent reservoir. If expanded cellular clones comprise a significant fraction of the latent reservoir, it should be possible to obtain from individual patients independent isolates of replication-competent virus with identical sequence throughout the viral genome. We were able to directly test this prediction in a unique way because of the large number of independent isolates of replication-competent HIV-1 obtained at limiting dilution after different rounds of stimulation in the MS-VOA. First, we amplified by RT-PCR the highly variable V3-V4 region of the *env* gene from viral RNA in supernatants of all p24^+^ wells from the MS-VOA from all 12 study subjects. Because the cells were initially plated at limiting dilution for viral outgrowth, most positive wells contained only a single sequence, and wells with multiple sequences were discarded. Sequences from each subject clustered together and separately from other subjects in phylogenetic analysis (Fig. S1). Although all participants started ART during chronic infection (Table S1), 9 of 12 subjects had one or more sets of independent isolates with identical sequence in the highly variable V3-V4 region of the *env* gene (Fig. 3). Sets of isolates with identical *env* sequences were seen in nine of nine patients from whom >10 isolates were obtained, strongly suggesting this phenomenon is general. It is important to note that isolates with identical *env* sequences are not the result of in vitro proliferation; all of these isolates originated from different wells of the original tissue culture plates (Fig. 1 b), indicating they are derived from different infected cells present in vivo. Of 197 independent isolates from 12 subjects, 113 (57%) belonged to sets of isolates with identical *env* sequences, whereas the remaining 43% had unique *env* sequences (Fig. 3).

### Isolates with identical *env* sequences are identical throughout the HIV-1 genome

**Figure 4.**
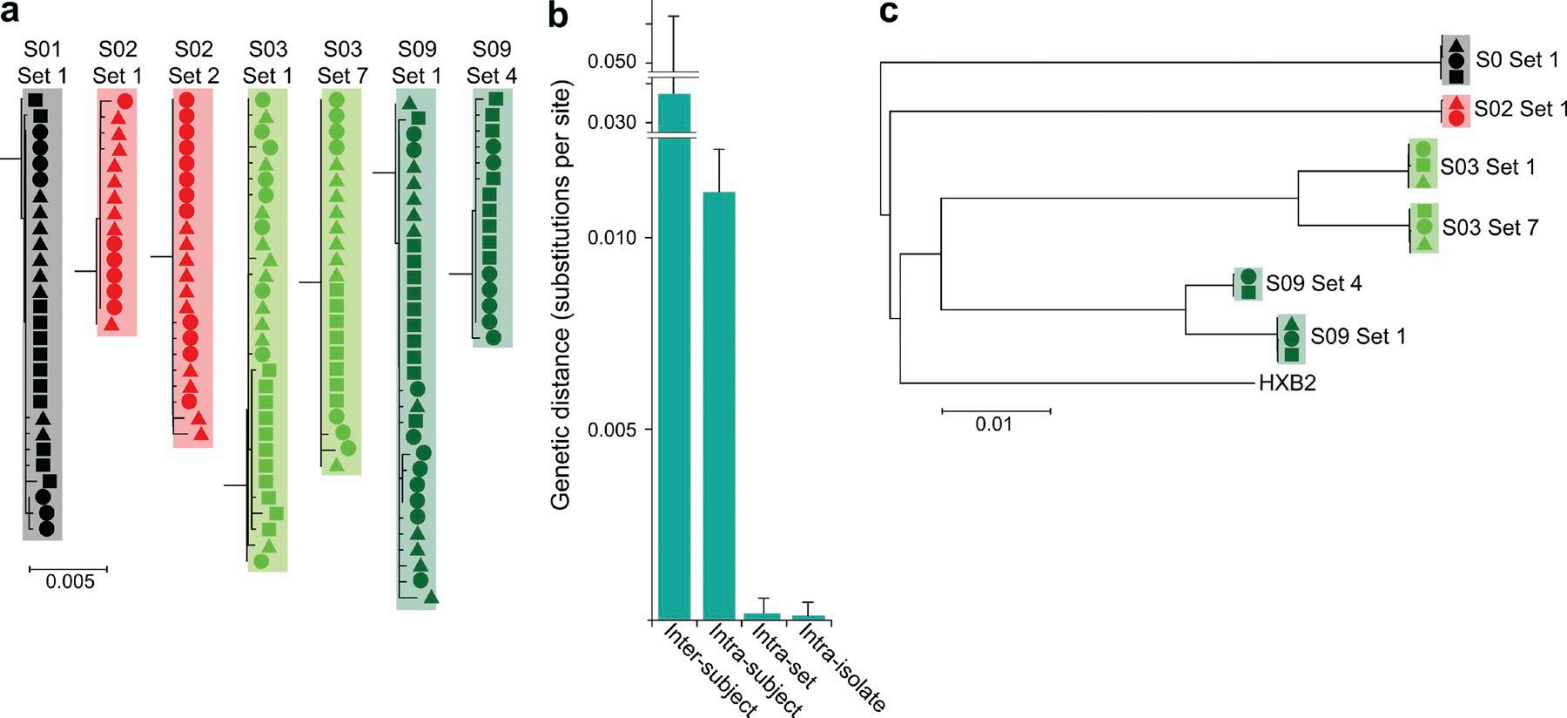
**Full-genome analysis of isolates with identical *env* sequences.** (a) Full-genome sequences of isolates with identical *env* sequences. Randomly selected isolates (arrows in Fig. 3) belonging to sets of isolates with identical *env* sequence were subjected to full-length sequencing. Sequencing was performed on single HIV-1 genomic RNA molecules present in the supernatants of p24^+^ cultures after reverse transcription and nested PCR amplification performed at limiting dilution. Two overlapping half-genome fragments were amplified. To minimize errors, PCR products were directly sequenced without cloning. For each isolate, 6–12 limiting 5′ sequences and 6–12 3′ sequences were obtained. Neighbor joining phylogenetic trees demonstrated that all sequences from a given set of isolates with identical *env* sequences co-clustered. The minimal differences (one to four nucleotides) likely reflect mutations expected to arise during the 3-wk outgrowth culture. Symbols indicate the PHA stimulation after which the isolates were detected, as in Fig. 3. Results are shown for the 5′ half-genome sequences. Similar results were obtained for the 3′ half-genome sequences. (b) Genetic distance (mean ± SD) between single-genome sequences from a single isolate (intra-isolate), between single-genome sequences from different isolates belonging to a set of isolates with identical *env* sequences (intra-set), between single-genome sequences from different sets from the same study subject (intra-subject), and between single genome sequences from different patients. For each set of two to three isolates from a given subject, 6–12 sequences were used for intra-isolate and intra-set comparisons. Then, all sequences were used for intra-subject and inter-subject comparisons. (c) Phylogenetic tree of full-genome consensus sequences. The 6–12 single half-genome sequences from each isolate were condensed to half-genome consensus sequences, which were joined to produce full-genome sequences. Set 2 from subject 2 is not depicted because of failure of the 3′ half-genome reaction as a result of primer mismatch.

These results are consistent with the idea that the majority of latently infected cells arise from proliferation of a smaller number of previously infected cells. However, alternative explanations must be excluded. First, it is possible that isolates with identical *env* sequences differ elsewhere in the genome (Laskey et al., 2016). To address this possibility, we first determined the clonal prediction score, a measure of the ability of subgenomic amplicons to predict clonality (Laskey et al., 2016). For this *env* amplicon, the clonal prediction score was 96, meaning that 96% of the time sequences identical in this region are identical throughout the entire viral genome based on available full-genome sequences from VOAs. To provide direct experimental evidence that isolates with identical *env* sequences were identical throughout the entire HIV-1 genome, we used single-genome analysis of genomic viral RNA in the supernatants of p24^+^ wells to obtain the full-genome sequences of representative isolates belonging to sets of isolates with identical *env* sequences. The sequencing was performed on two overlapping half-genome fragments. We were able to produce full-genome sequences from the half-genome sequences because the overlap regions were identical and, more importantly, because only a single provirus gave rise to outgrowth in these limiting dilution cultures. Although defective proviruses may have been present in these cultures, the profound nature of the commonly observed defects (Ho et al., 2013; Bruner et al., 2016; Imamichi et al., 2016) would preclude virion release into the supernatant. For each isolate tested, 6–12 single-genome sequences were obtained at limiting dilution from RNA in the supernatant. To avoid PCR errors, PCR products were sequenced directly without cloning (Hatziioannou et al., 2014). Sequences for each isolate were identical or very similar (Fig. 4 a), with one to four nucleotide differences likely representing the expected variation (Mansky and Temin, 1995) arising during the 3-wk culture period used to obtain each isolate (Fig. 1 b). Importantly, single-genome sequences from different isolates belonging to a given set with identical *env* sequences were intermingled in phylogenetic analysis (Fig. 4 a). The genetic distances between sequences from the same isolate and from other isolates belonging to the same set were not significantly different and were much smaller than the genetic distance between sequences from a given set and other isolates from the same subject (Fig. 4 b). The consensus half-genome sequences for each isolate were used to construct full-genome sequences. Phylogenetic analysis of full-genome sequences again showed that isolates with identical *env* sequences clustered together in a monophyletic pattern (Fig. 4 c). Together, these results demonstrate that isolates with identical *env* sequences are identical throughout the genome.

### Dominant viral sequence versus clonal expansion

**Figure 5.**
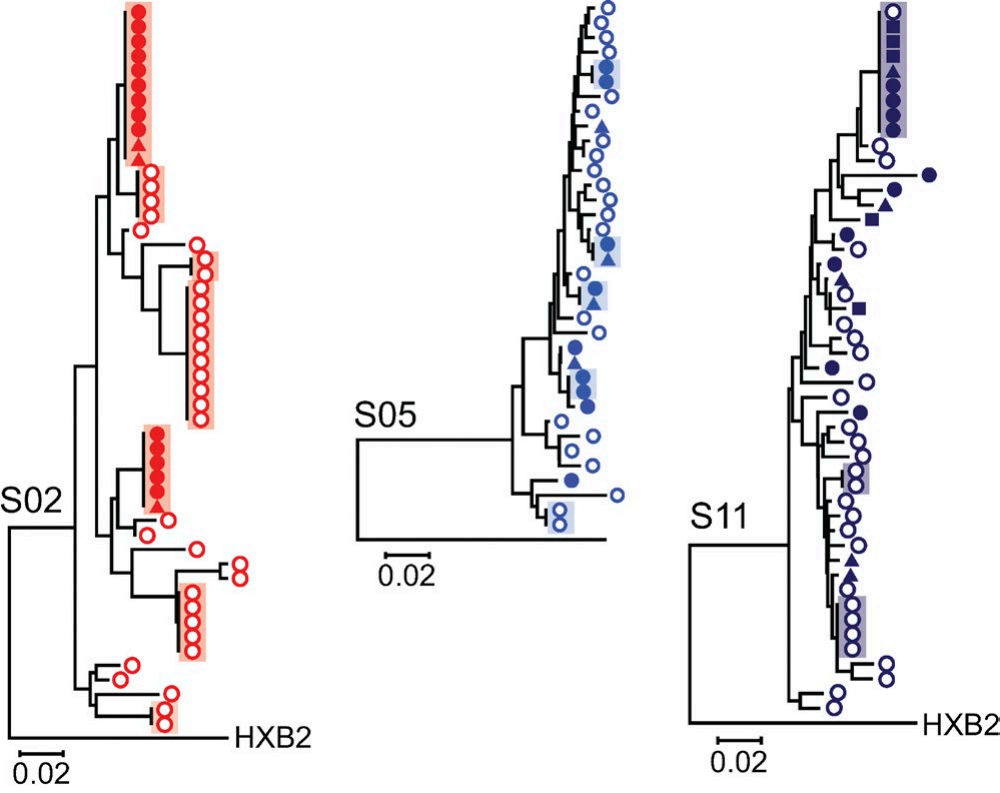
**Isolates with identical sequence are part of more complex proviral populations reflecting diversification over time during chronic infection.** Limiting dilution analysis of proviruses present in resting CD4^+^ T cells was performed on cells from three representative patients from whom sets of identical isolates of replication-competent virus were obtained. The V3-V4 region of *env* was sequenced, and the resulting sequences (open circles) were used in phylogenetic analysis along with the replication-competent isolates (closed symbol; shapes reflect the number of PHA stimulation as in Fig. 3). Sets of identical independent sequences are boxed.

There are two explanations for the presence in infected individuals of many infected cells with an identical viral sequence. Either all of the cells were infected by a predominant uniform virus population without mutation or a single cell carrying a particular viral sequence proliferated extensively after infection, copying the viral genome without error into daughter cells. The first explanation might apply during acute infection before diversification occurs. However, participants in this study initiated ART during chronic infection (Table S1) and, thus, are expected to have extensive viral sequence diversity (Shankarappa et al., 1999; Maldarelli et al., 2013; Brodin et al., 2016). To demonstrate this diversity, we used single-genome amplification of DNA from uncultured resting CD4^+^ T cells to obtain sequences of the *env* genes of proviruses from the same patients. These sequences were displayed on phylogenetic trees together with *env* sequences from the replication-competent isolates describe above. This analysis demonstrated that the replication-competent isolates were part of much more complex populations of proviruses in each subject (Fig. 5). Many of these proviruses may have defects elsewhere in the genome and thus will not necessarily show a close phylogenetic relationship to the replication-competent isolates. Together, these results show that the isolation of multiple independent clones of replication-competent virus with identical sequence is not caused by a lack of sequence diversity in these individuals. 

**Figure 6.**
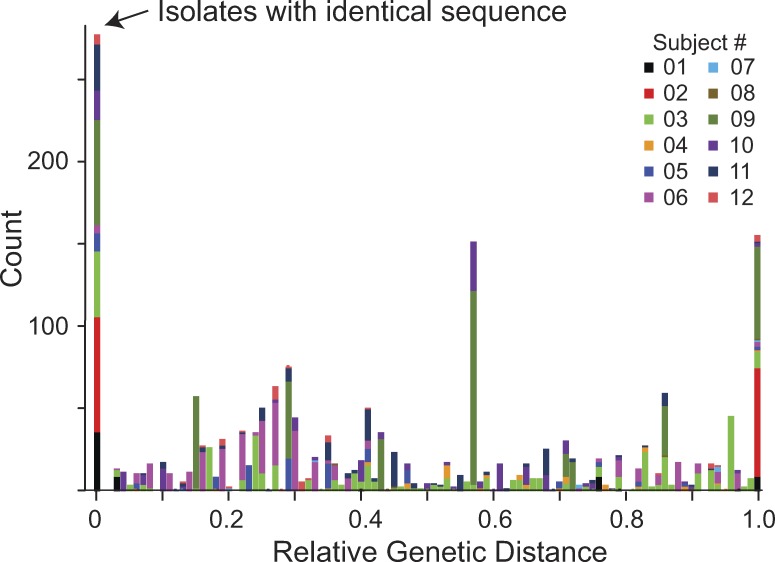
**Distribution of genetic distances between isolates of replication-competent virus from individual subjects (*n* = 12) does not support infection of a large number of cells by a dominant viral population.** Composite histogram showing the distribution of intra-subject genetic distances between isolates of replication-competent virus. To account for differing degrees of genetic divergence over time in different subjects, distances are normalized by the largest observed intra-subject distance. Analysis is based on the highly variable V3-V4 region of the *env* gene obtained as described in Fig. 3. The large peak at the origin reflects the zero branch lengths between independent identical isolates of replication-competent virus obtained from 9 of 12 patients.

To examine the possibility that isolates with identical sequence arose from infection of many cells by a dominant virus population, we first examined the genetic distance between isolates from the same subject (Fig. 6). This was done using the *env* sequences obtained from viral RNA in the supernatants of all clonal p24^+^ wells from the MS-VOA (Fig. 3). If within a given subject a substantial fraction of the latent reservoir is generated by infection of many cells by a dominant viral species, sequences close to the dominant species should also be present. Based on the error rate of reverse transcription (Mansky and Temin, 1995; Abram et al., 2010), a single base substitution is expected in 15–30% of cells infected by this dominant viral species. Other closely related sequences should be generated as this infection spreads, resulting in many sequences close to the dominant sequence. Therefore, we examined the distribution of genetic distances between independent isolates to determine whether isolates close in sequence to the dominant viral species were present. As is illustrated in Fig. 6, the observed distribution of intra-subject genetic distances does not support this hypothesis of infection of many cells by a dominant viral species. In fact, there is a striking paucity of variants close in sequence to the sets of identical sequences.

**Figure 7.**
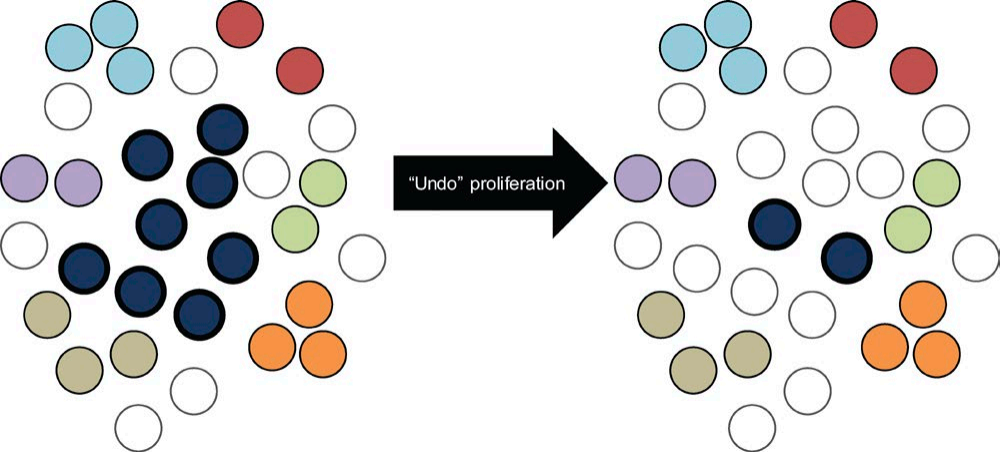
**Schematic illustration of a statistical test based on coalescent theory to explore the possibility that the high degree of sequence identity observed could be explained merely by rounds of viral replication in which no mutation occurred.** The left side of the figure represents the sample from participant 03 as analyzed by sequencing of the *env* gene in supernatant HIV-1 RNA from clonal p24^+^ wells (see Fig. 3). A total of 31 independent isolates were obtained, including one set with eight isolates (dark blue cells), six smaller sets (other colors), and eight unique isolates (singletons). The right side imagines a sample of the same size that would have been collected had no clonal proliferation occurred. The large clone is reduced to the size that maximizes likelihood under a neutral coalescent, and singletons are added to keep the total sample the same size. Because reducing the large clone essentially adds a parameter, a likelihood ratio test can be done. As shown in Table 1, p-values for all subjects tested in this manner were <0.05, indicating that mutation-free viral replication is an insufficient explanation for the data.

To further explore the possibility that the high fraction of independent isolates with identical sequence results from infection of many cells by a dominant viral species, we focused on five subjects (S01, S03, S10, S11, and S12) for whom there was a single large set of identical isolates that was phylogenetically separate from other isolates or sets of isolates from the same subject. We designed a statistical analysis based on coalescent theory to explore the possibility that the high degree of sequence identity observed could be explained merely by rounds of viral replication in which no mutation occurred. The data for the statistical test is the configuration of sets of identical isolates, that is, the number of sets of a given size containing identical *env* sequences (Table 1). For each patient, the mutation parameter estimate $\hat{\theta }$ is chosen to maximize the likelihood of observing the configuration according to Ewens’ sampling formula for the coalescent (Ewens, 1972). This calculation provides the nested model likelihood for the statistical test (i.e., likelihood without clonal proliferation). Clonal proliferation is assumed to alter the isolate set configuration by taking one sequence and increasing its multiplicity by a mechanism otherwise not present in the standard coalescent model. To model this behavior, we suppose that the proliferating sequence is (one of) the most frequent sequences observed and that it effectively replaced several singleton sequences that would have otherwise been observed in the sample. To maximize the likelihood, there are now two parameters: the mutation parameter (as in the nested model) and the number of sequences that this largest clone effectively replaced. We again used Ewens’ sampling formula but on an isolate set configuration that is modified to reverse the effect of this supposed replacement (Fig. 7). In other words, the largest clone is shrunk, and singleton sequences are added to keep the total sample size the same. The mutation parameter estimate $\hat{\theta }$ and log-likelihood are computed for this modified configuration. A p-value is then obtained using the likelihood ratio test with one degree of freedom (Table 1). The patterns in all five of these subjects significantly violate the distribution of isolate sets expected under a standard evolutionary dynamic without proliferation (P = 0.0003–0.027). For all five participants analyzed, the low p-values indicate that mutation-free viral replication is an insufficient explanation for the data.

**Table 1.** Statistical test for clonal proliferation

ID^a^	Actual isolate set configuration^b^				Modified isolate set configuration^c^			χ^2^ statistic (df = 1)	P-value^d^
	Size of isolate set/no. of sets of this size^e^	$\hat{\theta }$^f^	LL^g^		Size of isolate set/no. of sets of this size^h^	$\hat{\theta }$^f^	LL^g^		
01	1:3	1.67	−3.75		**1:12**	100	−0.64	6.23	*0.013*
	**9:1**								
03	1:8	10.81	−6.51		1:14	27.38	−4.06	4.91	*0.027*
	2:3				**2:4**				
	3:3				3:3				
	**8:1**								
10	1:8	11.74	−4.70		**1:14**	50.68	−1.85	5.71	*0.017*
	2:3				2:3				
	**6:1**								
11	1:11	12.96	−7.57		1:17	100	−1.08	12.98	*0.0003*
	**8:1**				**2:1**				
12	1:5	6.69	−3.01		**1:9**	100	−0.35	5.33	*0.021*
	**4:1**								

a Subjects with a single large set of identical isolate were included in the analysis.

b The nested model (i.e., model without clonal proliferation) uses the isolate set configuration observed for each subject.

c Clonal proliferation alters the configuration of sets of identical isolates by taking one sequence and increasing its multiplicity by a mechanism not present in the standard coalescent model. To model this behavior, we suppose that the proliferating sequence is the most frequent sequence observed and that it effectively replaces several singleton sequences that would have otherwise been observed in the sample. To maximize the likelihood, there are now two parameters: the mutation parameter (as in the nested model) and the number of sequences that the largest clone effectively replaced. We again use Ewens’ sampling formula but on an isolate set configuration that is modified to reverse the effect of this supposed replacement.

d P-value obtained using the likelihood ratio test with one degree of freedom. A value <0.05 (italicized) indicates that mutation-free viral replication is an insufficient explanation for the data.

e The largest set of isolates from each subject is shown in bold.

f For each subject, the mutation parameter estimate $\hat{\theta }$ is chosen to maximize the log-likelihood of observing the isolate set configuration according to Ewens’ sampling formula for the coalescent (Ewens, 1972). The maximum permitted value for $\hat{\theta }$ is 100.

g Log-likelihood.

h The size of the reduced isolate set that is created to reverse the effect of clonal proliferation is shown in bold.

Two simplifications may make this test too conservative. First, the test supposes that only one sequence may have been expanded. However, multiple sets of identical isolates were observed in several subjects. For example, subjects S02 and S09 both have two large sets of identical isolates that could have arisen through clonal proliferation. However, the statistical test was not designed to pick up this pattern. Second, by using only the allele configuration and not the full-sequence data, the test ignores other potential evidence for clonal proliferation. Note that the test checks for any mechanism that may increase the multiplicity of a sequence. For example, if positive selection before administration of ART dramatically increased the frequency of a sequence in the latent reservoir, it could produce a positive result by this test. However, in the setting of chronic infection, archival viral populations are highly diverse and mostly latent, making this sort of dramatic selective sweep unlikely. A final important simplification must be mentioned. The coalescent, used in this test, models a single contemporaneous sample of an evolving population experiencing generational turnover; however, the latent reservoir for HIV-1 is an archive populated over time, more closely resembling a longitudinal sample (Nickle et al., 2003). Overall, this analysis of the distribution of identical isolates is more consistent with clonal expansion of infected cells than with infection of multiple cells by a dominant viral population without mutation.

## Discussion

The extremely stable latent reservoir for HIV-1 in resting CD4^+^ T cells is a major barrier to cures (Finzi et al., 1999; Siliciano et al., 2003; Strain et al., 2003; Crooks et al., 2015). There is concern that the reservoir could be maintained or even expanded by cellular proliferation even when viral replication is fully arrested by ART. Proliferation of infected cells could be driven by antigens, cytokines, or effects related to the site of integration (Chomont et al., 2009; Maldarelli et al., 2014; Wagner et al., 2014). Previous sequencing studies have provided evidence for in vivo proliferation of infected cells (Tobin et al., 2005; Bailey et al., 2006; Imamichi et al., 2014; Maldarelli et al., 2014; Wagner et al., 2014; Cohn et al., 2015; von Stockenstrom et al., 2015; Lorenzi et al., 2016; Simonetti et al., 2016). However, a full understanding of the importance of clonal expansion requires consideration of three limitations of current studies. First, much of the evidence for clonal expansion comes from studies showing that multiple independent sequences from a single patient are identical. However, in most patients on ART, >98% of proviruses are defective (Ho et al., 2013; Bruner et al., 2016; Imamichi et al., 2016), and thus, without full-length sequencing (Chomont et al., 2009; Maldarelli et al., 2014; Wagner et al., 2014) or direct demonstration of viral replication (Lorenzi et al., 2016), it must be assumed that the identical sequences represent defective virus, particularly because cells carrying defective proviruses are less likely to die from viral cytopathic effects or lysis by host effector cells. Second, most studies use subgenomic amplicons, and it is possible that sequences identical in the regions analyzed differ elsewhere in the genome (Laskey et al., 2016). Most importantly, even complete viral sequence identity does not prove clonal expansion; an alternative explanation is infection of multiple cells by a dominant viral species. Integration site analysis provides direct evidence for clonal expansion but does not establish replication competence.

In light of these issues, two recent studies are of particular importance. Simonetti et al. have described an expanded cellular clone carrying replication-competent provirus integrated into a unique site in the human genome that could not be precisely mapped (Simonetti et al., 2016). This clone was identified in a patient with a concurrent malignancy that may have driven the clonal expansion (Simonetti et al., 2016). Lorenzi et al. recently demonstrated that 54% of replication-competent isolates from four treated patients had *env* sequences identical to other isolates from the same individual (Lorenzi et al., 2016). Together, these studies highlight the disturbing possibility that in vivo proliferation of infected cells may be a major factor in determining the composition and stability of the latent reservoir.

The data presented here contribute to our understanding of this issue in several ways. We directly demonstrate that cells carrying replication-competent HIV-1 can proliferate ex vivo without producing infectious virus and then release virus upon subsequent stimulation. Thus, strong mitogenic stimuli do not always reactivate latent HIV-1. In light of the short half-life of productively infected cells (Ho et al., 1995; Wei et al., 1995), this finding helps explain how clonal expansion could occur in vivo. We also demonstrate the presence of a large number of latently infected cells carrying identical viral sequences, such that cells with identical sequences can be routinely captured in a single blood sample from most patients. Overall, 57% of the isolates had *env* sequences identical to other isolates from the same patient. This finding agrees very well with the study by Lorenzi et al. (2016). We went on to show that sequences identical in the *env* gene were identical throughout the genome. In addition, through two independent approaches, we sought evidence that the identical sequences arose through infection of multiple cells by a dominant viral species. Both approaches failed to support this alternative hypothesis, leading us to favor the explanation that the identical sequences arose through the in vivo proliferation of latently infected cells. Definitive proof will require simultaneous identification of the integration site and demonstration of replication competence for many proviruses from multiple patients, something that cannot be readily achieved with current technology. Nevertheless, the experiments presented here raise the possibility that a substantial fraction of the latent reservoir is generated by cell proliferation, which our ex vivo experiments show can occur without release of infectious virus.

Our results have implications for measurement of the latent reservoir. Although PCR-based assays for proviral DNA are widely used, there is a large discrepancy between culture- and PCR-based assays of the latent reservoir. In comparative experiments, PCR assays for proviral DNA give infected cell frequencies that are ∼300-fold higher than frequencies measured by the QVOA (Eriksson et al., 2013). This is mainly because of the fact that the vast majority of proviruses are defective (Ho et al., 2013; Bruner et al., 2016; Imamichi et al., 2016). However, the number of proviruses that appear intact by full-length sequencing still exceeds the number induced to release infectious virus in the standard QVOA by 20–60-fold (Ho et al., 2013; Bruner et al., 2016). Here, we show using a multiple stimulation version of the QVOA that additional isolates of replication-competent HIV-1 can be obtained after the second, third, and fourth additional rounds of T cell activation. On average, only 60% of the isolates ultimately obtained in the MS-VOA grew out after a single stimulation. Thus, the standard QVOA and other assays that rely on a single round of T cell activation to induce latent HIV-1 may underestimate the frequency of latently infected cells, as previously suggested (Ho et al., 2013). It is important to determine what accounts for this difference. Some proviruses that appear intact by full-length sequencing may harbor minor missense mutations that decrease replication capacity. However, when apparently intact proviruses were reconstructed and tested for replication in primary CD4^+^ T lymphoblasts, six of six proviruses from four different infected individuals showed replication equivalent to that of NL4-3 and replication-competent viruses isolated from the same individuals (Ho et al., 2013). Another possibility is that some proviruses are permanently silenced by integration into regions nonpermissive for viral gene expression (Jordan et al., 2003; Lewinski et al., 2005), by transcriptional interference (Han et al., 2008; Lenasi et al., 2008; Shan et al., 2011), or by epigenetic modifications (Pearson et al., 2008; Blazkova et al., 2009; Tyagi et al., 2010). Finally, stochastic features of the Tat transactivation mechanism may prevent induction of all intact proviruses even in the setting of T cell activation (Razooky et al., 2015). Identification of successful latency-reversing strategies may require a deeper understanding of these issues.

The possibility that a substantial fraction of the latent reservoir arises by proliferation of a smaller number of infected cells presents some obvious challenges to HIV-1 cure efforts. It will be important to determine the factors driving the proliferation. In addition, because the total size of the reservoir does not increase over time (Finzi et al., 1999; Siliciano et al., 2003; Crooks et al., 2015), any proliferation must be roughly balanced by cell loss, and it will be important to understand the mechanisms involved and whether they can be enhanced to promote cure.

## Materials and methods

### Study subjects

Subjects were HIV-1–infected adults who met the inclusion criteria of suppression of viremia to <20 copies HIV-1 RNA/ml of plasma on ART for >6 mo. This study was approved by the Johns Hopkins Institutional Review Board. Written informed consent was obtained from all subjects.

### MS-VOA

The MS-VOA was performed on purified resting CD4^+^ T cells as described previously (Ho et al., 2013; Laird et al., 2013, 2016) with modifications to allow four consecutive stimulations with PHA (Fig. 1, a and b). Resting CD4^+^ T cells were isolated from PBMCs using a two-step negative selection protocol (Laird et al., 2016) with monoclonal antibodies and magnetic beads (Miltenyi Biotec) as previously described and plated in the upper chambers of 12-well transwell plates (Corning) at a predetermined limiting dilution for viral outgrowth (200,000 cells per well). Cells were activated with 0.5 µg/ml PHA and irradiated allogeneic PBMCs from normal donors as previously described (Finzi et al., 1997, 1999; Siliciano et al., 2003). The next day, half the media from each transwell was removed and replaced with fresh media lacking PHA, and then, 10^6^ MOLT-4/CCR5 cells were added to the bottom chamber of the transwell to allow robust replication of virus released from infected cells. A previous study has shown that viral outgrowth in this transwell system is equivalent to that seen with direct co-culture (Ho et al., 2013). MOLT-4/CCR5 cells were obtained from the National Institutes of Health AIDS Reagent Program and were maintained in G418 until use in the assay. The cells were tested for CCR5 expression by flow cytometry and were negative for mycoplasma. 8 d after the initial PHA stimulation, half of the volume from both the top and bottom chambers of each transwell was transferred to a new set of transwell plates for a second round of PHA stimulation. The initial plates were cultured without further stimulation for a total of 21 d. The restimulated plates were cultured for 8 d after the second round of PHA stimulation and then split as above to generate a third set of plates, which received a third round of stimulation. Similarly, these plates were split 8 d later to generate the fourth set of plates, which received a fourth round of stimulation. A p24 ELISA (PerkinElmer) was performed on the supernatant 21 d after each respective round of PHA stimulation (Fig. 1 b).

### CFSE dilution and activation/exhaustion marker staining

An aliquot of resting CD4^+^ T cells was stained with 5 µM CFSE before the initial PHA stimulation. The dilution of CFSE was analyzed 1 wk later by flow cytometry using a 488-nm laser on a FACSCanto II cytometer (BD) with an emission of 492/517 nm. Unstimulated cells served as a control. Expression of activation markers was analyzed 1 wk after each round of PHA stimulation. An aliquot of cells was stained with anti-CD4 (FITC), anti-CD25 (APC), anti-CD69 (APC), and anti–HLA-DR (APC) antibodies (BioLegend) at 4°C for 15–30 min and analyzed by flow cytometry on the FACSCanto II cytometer. Cells that did not receive the most recent round of PHA stimulation served as controls. To assess changes in expression of inhibitory receptors, cells were stained 1 wk after each round of PHA activation with anti-PD1 (FITC), anti–CTLA-4 (PE), and anti–Tim-3 (P3/Cy7) antibodies (BioLegend) and analyzed by flow cytometry.

### RNA isolation, cDNA synthesis, and amplification of the env gene

Viral RNA was isolated from 200 µl of the supernatant from each p24^+^ well using a ZR-96 Viral RNA kit (Zymo Research Corporation). Then, RNA was treated with DNase (Thermo Fisher Scientific) and reverse transcribed using the qScript cDNA Supermix kit (Quanta Biosciences). Because the cultures were seeded at limiting dilution for viral outgrowth, we ran a nested PCR on undiluted cDNA from each well. A nested PCR for the V3-V4 region of *env* was performed using 600 ng cDNA and primers ES7 (5′-CTGTTAAATGGCAGTCTAGC-3′) and ES8 (5′-CACTTCTCCAATTGTCCCTCA-3′) for the outer reaction. The outer PCR products were diluted 1:50, and 5 µl of this dilution was used for the inner PCR reaction with primers Nesty8 (5′-CATACATTGCTTTTCCTACT-3′) and DLoop (5′-GTCTAGCAGAAGAAGAGG-3′). Primers were obtained from Integrated DNA Technologies. Amplification conditions were as follows: denaturation at 94°C for 3 min, followed by 40 cycles of denaturation at 94°C for 30 s; annealing at 55°C for 30 s; and extension at 68°C for 5 min. PCR products were run on a 1% agarose gel, and bands were extracted using the QIAquick Gel Extraction kit (QIAGEN). Extracted DNA was analyzed directly by Sanger sequencing at Genewiz, Inc.

### Amplification of the env gene from proviral DNA

Nested PCR performed at limiting dilution was used to analyze the *env* gene of proviruses in resting CD4^+^ T cells from study subjects. Amplification was performed as previously described (Bailey et al., 2006), except the outer PCR primers were the ES7/ES8 described in the previous paragraph. 1 µl of the outer PCR product was used for the inner PCR reaction, with the Nesty8/DLoop primers described in the previous paragraph. PCR products were directly sequenced by Sanger sequencing.

### Full-genome sequencing

For representative isolates, viral RNA was sequenced by single-genome amplification as previously described (Hatziioannou et al., 2014). Viral RNA in the supernatants of p24^+^ wells was extracted and reverse transcribed. cDNA was serially diluted and amplified with nested PCRs in two overlapping half-genome reactions as previously described (Li et al., 2010). For each isolate, 6–12 amplicons obtained at the limiting dilution were directly sequenced, and a full-genome consensus sequence was generated as described in the Isolates with identical *env* sequences are identical throughout the HIV-1 genome section of Results.

### Phylogenetic analysis

Forward and reverse sequences for each sample were aligned into a single consensus contig per sample using default assembly parameters on CodonCode Aligner software (CodonCode Corporation). Rare sequences that did not appear clonal were discarded. Each sample consensus sequence was aligned with reference sequences of cataloged viruses from the Los Alamos National Laboratory HIV sequence database using default assembly parameters that were adjusted to accommodate all sample and reference sequences. For phylogenetic tree generation, sequences were trimmed to the same length. Genetic distances were calculated, and neighbor joining trees (Tamura et al., 2004) were generated using a maximum composite likelihood algorithm and default parameters using MEGA7 software (Molecular Evolutionary Genetics Analysis Program; Kumar et al., 2016). Maximum likelihood trees were also generated. The conclusions were not sensitive to the method of tree generation, and neighbor joining trees are shown in the figures.

### Data availability

Sequences are available through GenBank under accession nos. KY780963–KY781160.

### Statistical test for clonal proliferation

We designed a statistical analysis based on coalescent theory to explore the possibility that the high degree of sequence identity observed could be explained merely by rounds of viral replication in which no mutation occurred. The test was applied to a subset of study participants (01, 03, 10, 11, and 12) for whom a single large set of identical isolates were phylogenetically distinct from smaller sets and unique individual sequences (singletons) from the same participant (Fig. 3). For the other participants, the test could not be readily applied because there were no singletons (02), no gap between small and large sets (05 and 06), or no unique large clone after the gap (09). For each patient, the mutation parameter estimate $\hat{\theta }$ was chosen to maximize the likelihood of observing the configuration according to Ewens’ sampling formula for the coalescent (Ewens, 1972). This calculation provides the nested model likelihood for the statistical test (i.e., likelihood without clonal proliferation). Clonal proliferation is assumed to alter the isolate set configuration by taking one sequence and increasing its multiplicity by a mechanismnot present in the standard coalescent model. To model this behavior, we suppose that the proliferating sequence is (one of) the most frequent sequences observed and that it effectively replaced several singleton sequences that would have otherwise been observed in the sample. To maximize the likelihood, there are now two parameters: the mutation parameter (as in the nested model) and the number of sequences that this largest clone effectively replaced. We again use Ewens’ sampling formula but on an isolate set configuration that is modified to reverse the effect of this supposed replacement. In other words, the largest clone is shrunk, and singleton sequences are added to keep the total sample size the same. The mutation parameter estimate $\hat{\theta }$ and log-likelihood are computed for this modified configuration. A p-value is obtained using the likelihood ratio test with one degree of freedom.

### Online supplemental material

Fig. S1 shows a phylogenetic tree of *env* V3-V4 sequences from independent isolates of replication-competent HIV-1 from resting CD4^+^ T cells from 12 patients on ART. Table S1 lists characteristics of study subjects.

## Supplementary Material

Supplemental Materials (PDF)
