# Supplementary material for: Proliferation of latently infected CD4+ T cells carrying replication-competent HIV-1: Potential role in latent reservoir dynamics
Source: J Exp Med. 2017 Apr 3;214(4):959–72. doi: 10.1084/jem.20170193 (PMC5379987; doi:10.1084/jem.20170193)
Supplement: Supplemental Materials (PDF) [file JEM_20170193_sm.pdf]

The Journal of Experimental Medicine

SUPPLEMENTAL MATERIAL

Hosmane et al., https://doi.org/10.1084/jem.20170193

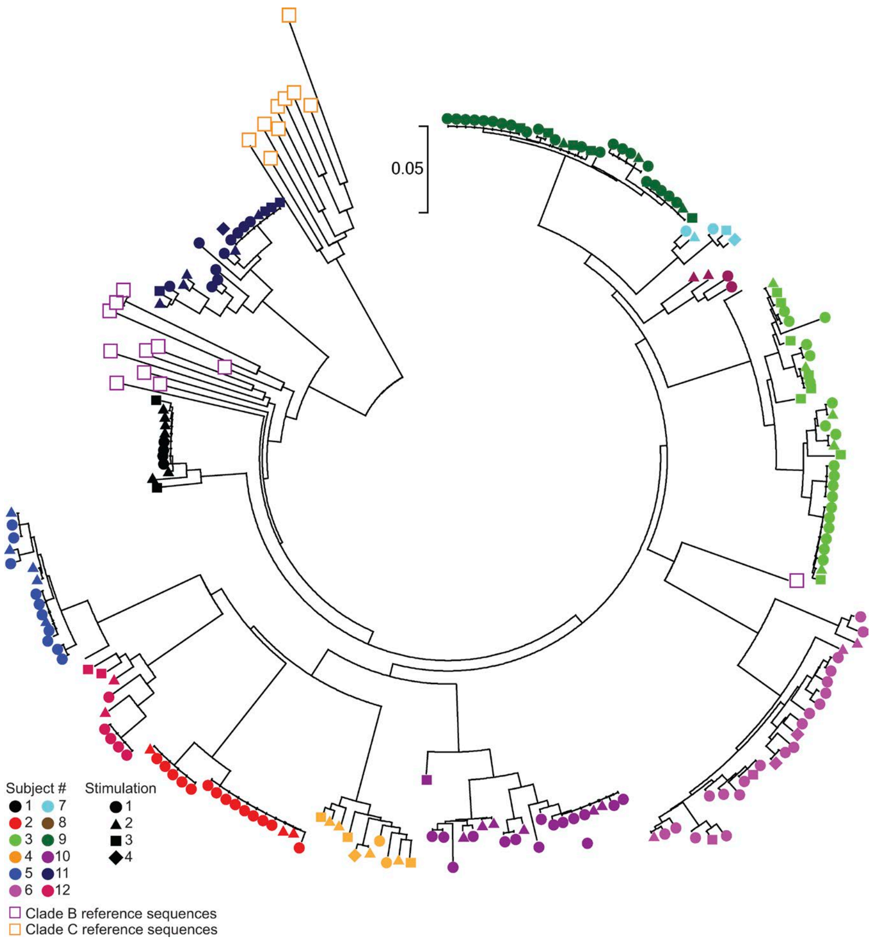

Figure S1. **Phylogenetic tree of *env* V3–V4 sequences from independent isolates of replication-competent HIV-1 from resting CD4⁺ T cells from 12 patients on ART.** Each filled symbol represents an independent isolate obtained in the MS-VOA. Symbol shape indicates the number of PHA stimulations after which the isolate was detected. Open symbols are clade B and C reference sequences.

Table S1. **Characteristics of study subjects**

ID[a]	Sex	Year HIV$^+$	Earliest CD4 count		Earliest plasma HIV-1 RNA		Times before sampling			Values at sampling		
			Year	Value	Year	Value	Time HIV$^+$	Time on ART	Time with VL <50 copies/ml	CD4 count	Viral load	ART regimen
				cells/µl		*copies/ml*	*yr*	*yr*	*yr*	*cells/µl*	*copies/ml*	
S01	M	1996	1997	239	1997	ND[b]	17.9	17.8	15.8	682	<20	ABC/3TC, DRV/r
S02	F	1995	1995	392	1996	2,712	19.4	7.4	6.5	1,115	<20	ETR, RAL, DRV/r
S03	F	2004	2005	186	2005	11,348	9.8	6.6	5.7	624	<20	FTC/TDF, ATV/r
S04	F	2003	2003	294	2003	>750,000	11.1	2.5	2.3	450	<20	RAL, MVC, DRV/r
S05	F	1999	1999	346	1999	12,717	15.3	15.2	14.7	800	<20	ABC/3TC, NVP
S06	M	1994	1996	820	1996	4,203	20.9	7.7	7.3	1,478	<20	EFV/FTC/TDF
S07	M	1999	2003	14	2003	742,939	16.1	11.8	11.3	706	<20	FTC/RPV/TDF, TDF
S08	M	1986	2010	734	2010	<50	29.3	4.6	4.3	865	<20	EFV/FTC/TDF
S09	M	1992	2000	670	2000	ND	23.3	23.2	15.1	1,156	<20	ABC, 3TC, ETR, RAL
S10	F	1998	2007	61	2007	117,375	17.4	8.3	4.8	771	<20	FTC, LPV/r, RAL
S11	M	2007	2007	500	2007	140,000	8.4	3.9	2.5	962	<20	TDF/FTC, DTG
S12	F	1990	1995	504	1997	3,952	25.7	6.4	6.2	980	<20	RAL, MVC, DRV/r

Abbreviations used: 3TC, lamivudine; ABC, abacavir; ATV, atazanavir; DRV, darunavir; DTG, dolutegravir; EFV, efavirenz; ETR, etravirine; F, female; FTC, emtricitabine; LPV, lopinavir; M, male; MVC, maraviroc; NVP, nevirapine; r, ritonavir-boosted; RAL, raltegravir; RPV, rilpivirine; TDF, tenofovir.

[a]Participants were selected based only on CD4 count >200 cells/µL and prolonged (>6 mo) suppression of viremia on ART.

[b]ND, none detected; lab result negative for presence of HIV-1 RNA in blood.
